# Suitability of Human Mesenchymal Stem Cells Derived from Fetal Umbilical Cord (Wharton’s Jelly) as an Alternative In Vitro Model for Acute Drug Toxicity Screening

**DOI:** 10.3390/cells11071102

**Published:** 2022-03-24

**Authors:** Ioannis Christodoulou, Maria Goulielmaki, Andreas Kritikos, Panagiotis Zoumpourlis, Georgios Koliakos, Vassilis Zoumpourlis

**Affiliations:** 1 Biomedical Applications Unit, Institute of Chemical Biology, National Hellenic Research Foundation (NHRF), 11635 Athens, Greece; christodoulou.ioannis@gmail.com (I.C.); mgoulielmaki@eie.gr (M.G.); perkizitore@gmail.com (A.K.); stm02506@uoi.gr (P.Z.); 2 Cancer Immunology and Immunotherapy Center, Saint Savas Cancer Hospital, 11522 Athens, Greece; 3 Medical School, Aristotle University of Thessaloniki, 54124 Thessaloniki, Greece; koliakos@biohellenika.gr

**Keywords:** Wharton’s jelly, human mesenchymal stem cells, in vitro cytotoxicity, acute drug toxicity screening, 3D (three-dimensional) culture

## Abstract

Preclinical toxicity screening is the first and most crucial test that assesses the safety of new candidate drugs before their consideration for further evaluation in clinical trials. In vitro drug screening using stem cells has lately arisen as a promising alternative to the “gold standard” of animal testing, but their suitability and performance characteristics in toxicological studies have so far not been comprehensively investigated. In this study, we focused on the evaluation of human mesenchymal stem cells isolated from the matrix (Wharton’s jelly) of fetal umbilical cord (WJSCs), which bear enhanced in vitro applicability due to their unique biological characteristics. In order to determine their suitability for drug-related cytotoxicity assessment, we adopted a high-throughput methodology that evaluated their sensitivity to a selected panel of chemicals in different culture environments. Cytotoxicity was measured within 48 h by means of MTS and/or NRU viability assays, and was compared directly (in vitro) or indirectly (in silico) to adult human mesenchymal stem cells and to reference cell lines of human and murine origin. Our data clearly suggest that human WJSCs can serve as a robust in vitro alternative for acute drug toxicity screening by uniquely combining rapid and versatile assay setup with high-throughput analysis, good representation of human toxicology, high reproducibility, and low cost.

## 1. Introduction

Toxicity testing is an integral part in the development process of any new product destined for human consumption or use—most notably those stemming from the pharmaceutical, chemical, agrochemical, and medical device industries. In drug discovery, for example, synthesizing and screening a huge number of candidate molecules is often required before finding the ultimate active compound that has maximal pharmacological effects and minimal adverse and toxic effects [1]. Toxicity testing in the preclinical stage is crucial, since it provides the dosing data on which the human clinical trials are based; nevertheless, it is an expensive and time-consuming process that relies heavily on the use of animals [2]. In the EU alone, in a given year, more than 150,000 animals (mostly rodents) are used, at a cost of almost EUR 19 million, just for acute toxicity screening purposes; the number of animals can rise up to 1 million in the case of comprehensive toxicity studies [3]. This massive use of animals not only raises major ethical issues, but is also largely inefficient, since the significant biological differences and evolutionary diversification between small mammals and humans does not allow a reliable extrapolation of toxicity data from the former to the latter, therefore severely limiting the predictive capacity of animals for toxicity evaluation.

As a response to all of these issues, international regulation authorities have advocated the establishment of (in vitro) alternative drug toxicity testing systems that will reduce, refine, and replace extensive animal testing, and that will be able to predict human toxicity reliably and economically [4]. Possible alternatives to the “gold standard” of animal testing include in vitro screening using human primary cells, continuous cell lines of human or animal origin and, in the last decade, stem cells.

In 2006, the Interagency Coordinating Committee on the Validation of Alternative Methods (ICCVAM) validated two cell lines as standards for the prediction of acute in vitro cytotoxicity: BALB/c 3T3 murine fibroblasts, and normal human keratinocytes (NHKs) [5,6,7]. The proposed procedure for characterizing a cell type as suitable for predicting in vitro cytotoxicity involves testing at least 12 chemicals that cover all 5 hazard categories of the Globally Harmonized System (GHS) of Classification and Labelling of Chemicals for acute oral toxicity [8]. This is followed by a linear correlation of the IC_50_ values with LD_50_ values from the Registry of Cytotoxicity (RC), using linear regression [9]. In the case of the two abovementioned validated cell lines, two new equations were also used—the RC rat-only millimole, and the RC rat-only weight—for the prediction of oral LD_50_ values. Cell viability was determined using the neutral red uptake (NRU) assay.

Although reliable for toxicity testing, BALB/c 3T3 and NHK cells are not adequately accurate for safely determining the acute oral toxicity of chemicals. The need for novel in vitro cytotoxicity models, which can be used universally as a cellular platform for accurately predicting acute toxicity, remains. Immortalized or transformed cell lines do not constitute a plausible replacement option, since they differ significantly from their respective non-transformed cells, in terms of both physiology and function; therefore, the obtained results cannot safely be translated to corresponding consequences for healthy human tissues [10]. With respect to human primary cells, culturing these poses significant technical complications and limitations, including the difficulty of isolating considerable cell numbers and the short lifespan of these cells in vitro [11]. In recent years, stem cells have been dynamically introduced into toxicological studies due to their unique characteristics; these include the capacity for long-term in vitro propagation and differentiation into specialized cell types—a property that further enables their exploitation for developmental and functional toxicity testing [12,13]. Bone-marrow-derived mesenchymal stem cells (BMSCs) have been evaluated according to the ICCVAM protocol, and have been proposed as a new, reliable alternative to the already validated cell models [14]; however, there are limitations regarding the use of these cells in toxicity testing, including isolation difficulty, donor heterogeneity, and early senescence during cultivation [15,16].

Mesenchymal stem cells isolated from Wharton’s jelly (WJSCs) within the human umbilical cord carry obvious advantages compared to adult cells, rendering them an attractive choice for use in in vitro toxicity assays; these include their practically inexhaustible source, safe and inexpensive isolation, easy ex vivo propagation, low immunogenicity, and high phenotypic and genetic stability in culture [17,18]. The aim of the present study was to test the ability of human fetal MSC (WJSC)-based basal cytotoxicity assays to correctly predict LD_50_ and the hazard category according to the GHS. The approach adopted was based on the regression model developed by Spielmann et al. [19], following the rules established by ICCVAM after the BALB/c 3T3 and NHK validation studies [5,6]. The ICCVAM recommendations require that any new cell line to be tested should be demonstrated to meet or exceed the accuracy and reliability of the two already validated cell lines BALB/c 3T3 and NHK. Towards this end, we adopted a 96-well plate high-throughput screening (HTS) platform on which IC_50_ was determined by end-point assays by measuring viability in WSJCs grown in different culture environments, and following 48 h exposure to various concentrations of a selected panel of chemicals. We then evaluated the performance of the model by cross-validation comparison of the data generated against other cytotoxicity assays, both in vitro and in silico.

## 2. Materials and Methods

### 2.1. Chemicals

All chemicals were purchased from Sigma-Aldrich (Saint Louis, MO, USA). Ultimately, 12 substances were used out of a total of 30 reference compounds—2 for each of the 5 GHS risk categories, and 2 unclassified ones, according to the guidelines set by the ICCVAM [5,6]. The toxicity of the selected compounds ranged from LD_50_ ≤ 5 mg/kg (hazard category 1) to LD_50_ > 5000 mg/kg (hazard category 6). Stocks and serial dilutions were prepared according to the manufacturers’ instructions and the ICCVAM report [6]. Sodium dodecyl sulfate (SDS) was used as a positive control. All chemicals were handled using the necessary precautions dictated by the material safety datasheet (MSDS) provided by the manufacturer.

### 2.2. Cells Lines

For in vitro assays, four different cell types were used. HepG2 (human hepatocellular carcinoma cell line) and NIH 3T3 (murine embryonic fibroblast cell line) cells were both purchased from the ECACC. Human adipose-tissue-derived mesenchymal stem cells (ADSCs) were previously isolated from abdominal fat aspirates of patients undergoing voluntary liposuction surgery [18]. Human Wharton’s-jelly-derived mesenchymal stem cells (WJSCs) were previously isolated from the matrix of the umbilical cord from full-term pregnancies [18]. Both cell types were characterized for the expression of surface markers via flow cytometry, and were positive for CD29 (b1-integrin), CD44 (H-CAM), CD73 (ECTO-5’nuclease/SH3), CD90 (THY-1), and CD105 (endoglin/SH2), and negative for CD14 (LeuM3/MY4), CD34 (HPCA1/gp105-120), and CD45 (LCA) [18].

### 2.3. Cell Culture

WJSCs up to the 7th passage (<22 population doublings) and ADSCs up to the 5th passage (<6 population doublings) were used for the experiments. At these culture points, WJSCs and ADSCs maintained a stable mesenchymal stem cell (MSC) phenotype, a typical MSC immunophenotypic profile—as previously described—and a mean population doubling time (PDT) of 32 h and 8 days, respectively [18]. Cells were propagated in culture as previously described [18]. Briefly, cells were plated in flasks of 75 cm^2^ (Corning) and cultured in growth medium (GM), which consisted of DMEM/F12 (with 3.5 g/L glucose, UltraGlutamine I, and Na pyruvate; Lonza, Basel, Switzerland) supplemented with 10% fetal bovine serum (FBS), 15 mM HEPES, 1× nonessential amino acids, 1% penicillin/streptomycin, and 2 mM Fungizone (all from Invitrogen, Carlsbad, CA, USA). Frozen aliquots of 0.5 − 2 × 10^6^ cells in 2 mL of 10% DMSO in FBS were stored in cryovials (Nunc, Rochester, NY, USA), in liquid N_2_. NIH 3T3 and HepG2 cells were seeded in flasks of 75 cm^2^ in aMEM culture medium (supplemented with 10% FBS, 1% penicillin/streptomycin, and 2 mM L-glutamine; Sigma, Saint Louis, MO, USA). Frozen aliquots of 0.5 − 2 × 10^6^ cells in 2 mL of 50% FBS, 40% aMEM, and 10% DMSO were stored in cryovials (Nunc), in liquid N_2_.

The cells were maintained in a humidified atmosphere with 5% CO_2_ in air at 37 °C, with medium changes every 3–4 days, until 70–80% confluence. Sub-culturing (passages) was performed by trypsinization using 0.05% trypsin–EDTA solution (Invitrogen) and new cell plating at a density of 4000 cells/cm^2^ in flasks of 75 cm^2^.

### 2.4. D-Cell Culture of WJSCs

Ex vivo culture of WJSCs in three-dimensional (3D) conditions was performed using scaffolds with a structure of a polystyrene microfiber network (3D InsertTM-PS scaffolds, Sigma-Biotek, Saint Louis, MO, USA). The scaffold was in the form of discs that fit as inserts into the wells of 96-well microtiter plates. The optimal seeding density was determined by running the MTS assay with different cell densities (5, 6, 10, 15, or 20 × 10^3^ cells) to determine the number of cells that would enable an exponential (LOG) growth phase during testing.

Cells (15 × 10^3^ WJSCs) resuspended in 15 μL of GM were carefully seeded on the center of the discs’ surface in quadruplicate and incubated in 5% CO_2_ in air, at 37 °C, for 3 h. Each well was then supplemented with another 185 μL of GM and cells were left to grow for 48 h. At the end of this incubation period, media were replaced with GM containing test chemicals at various concentrations for the determination of cytotoxicity/cell viability, as described below.

### 2.5. Determination of Viability/Cytotoxicity 

Cell viability was determined colorimetrically with a 96-well plate assay using either the tetrazolium-based MTS assay or the neutral red uptake (NRU) assay. For both assays, WJSCs or ADSCs were plated in triplicate into 96-well microtiter plates at a concentration of 3.2 × 10^3^ or 5 × 10^3^ cells, respectively, in 100 μL of GM. For serum-free cytotoxicity experiments, WJSCs were plated in triplicate into 96-well microtiter plates, in 100 μL of GM, without the addition of FBS. NIH 3T3 and HepG2 cells were plated at concentrations of 2.5 × 10^3^ and 5 × 10^3^ cells, respectively, in 100 μL of GM. Cells were then incubated (37 °C/5% CO_2_) for 48 h to ensure sufficient cell recovery and adhesion. For WJSCs, the optimal seeding density was determined by running the MTS assay with different cell densities (1.5, 2, 2.5, 3, 3.5, 4, and 4.5 × 10^3^ cells) to determine the number of cells that would enable an exponential (LOG) growth phase during testing. After the 48 h incubation, the media were removed and the cells were treated with 8 different concentrations of each of the test chemicals in 100 μL of medium (aMEM for HepG2 and NIH 3T3, or DMEM/F12 for WJSCs and ADSCs). All chemicals were directly dissolved in the respective GM without any solvent. Cells serving as a negative control were incubated in plain culture medium. Cells were then incubated for 48 more hours.

### 2.6. MTS Assay

The MTS (CellTiter 96^®^ AQueous One, Promega, Fitchburg, WI, USA) assay was performed following the manufacturer’s recommendations. The tetrazolium compound MTS was bio-reduced by cells into a colored formazan product that is soluble in tissue culture medium [20]. This conversion was presumably accomplished by NADPH or NADH produced by dehydrogenase enzymes in metabolically active (viable) cells.

After the incubation period, media containing the chemicals were removed from all of the wells, and cells were washed with 150 μL/well of prewarmed PBS. In turn, 100 μL of DMEM/F-12 (without phenol red, L-glutamine, or HEPES) was added to each well, in order to eliminate the absorbance signal given by the pH indicator; 20 μL of CellTiter 96 ^®^ AQueous One Solution Reagent (Promega, Fitchburg, WI, USA) per well was also added. The CellTiter 96 ^®^ AQueous One Solution Cell Proliferation Assay is a colorimetric method for determining the number of viable cells in proliferation assays or cytotoxicity. To blank wells, PBS was added. The cells were incubated for another 4 h. Finally, absorbance was detected at 490 nm (as well as at 650 for noise elimination) using a monochromator microplate reader safire2 (Tecan Austria GmbH, Salzburg, Austria)/ measurement parameter editor Magellan (version 6).

### 2.7. Neutral Red Uptake (NRU) Assay

The NRU (Sigma-Aldrich, Saint Louis, MO, USA) assay was performed following the research protocol proposed by Borenfreund and Puerner [21]. This method is based on the ability of living cells to internalize and bind the neutral red dye (toluene red). Neutral red (NR) readily penetrates the cell membranes of living cells via non-ionic diffusion, and accumulates intracellularly in the lysosomes. Alterations induced by chemicals on the cell membrane or on the sensitive membranes of lysosomes lead to reduced uptake and binding of NR.

Briefly, after the incubation period, media containing the chemicals were removed from all of the wells, and cells were washed with 150 μL/well of prewarmed PBS. In turn, 250 μL of neutral red medium (1 mL of NR stock solution, 79 mL of αMEM; stock solution: 0.4 g of NR dye, 100 mL of Milli-Q H_2_O) was added to each well and incubated for 3 h. Then, the NR medium was removed, and the cells were rinsed with 250 μL/well of prewarmed PBS. After elution of the dye in 100 μL/well of NR desorbing fixative (1% glacial acetic acid solution, 50% ethanol, 49% H_2_O), the plate was shaken for 20 min in the dark. Finally, absorbance was detected at 540 nm (as well as at 690 for noise elimination) using a monochromator microplate reader safire2 (Tecan Austria GmbH, Salzburg, Austria)/measurement parameter editor Magellan (version 6).

### 2.8. Cell Imaging

Two-dimensional cultures of cells were observed under an Olympus inverted microscope, and images were captured with an on-board CCD camera. For immunofluorescence staining of the cell-seeded 3D constructs, the cell cytoskeleton was stained in situ with phalloidin–FITC (Invitrogen) after cell fixation with 4% paraformaldehyde (PFA, Sigma, Saint Louis, MO, USA) and permeabilization with 0.25% Triton-X, and cell nuclei were counterstained with DAPI (Sigma, Saint Louis, MO, USA). Photographs of the stained 2D cultures were taken under a confocal laser scanning microscope (Leica 626 TCS SPE). LAS AF software was used for image acquisition (Leica Lasertechnik, Heidelberg, Germany).

### 2.9. Statistical Analysis

Calculation of IC_50_ values, correlation analysis (F-test), linear regression, and ANOVA were performed using GraphPad Prism 5.0.3.

Absorbance values from the microtiter plate reader were transferred to a Microsoft Office Excel 2010 ^®^ spreadsheet template to determine % cell viability (compared to corresponding negative controls), as well as to verify the test acceptance criteria established by ICCVAM [6]. In detail, the IC_50_ values for each substance were calculated using the following rearranged Hill function, i.e., a four-parameter (sigmoidal) logistic mathematical model, by means of GraphPad Prism 5.0.3 statistical software:

log IC_50_ = log EC_50_ − {[log(Top-Bottom/Y-Bottom) − 1]/HillSlope} (1)

where IC_50_ is the concentration causing 50% reduction in cell viability, EC_50_ is the concentration causing a response midway between the Top and Bottom responses, Top is the maximal response (maximal viability, i.e., 100), Bottom is the minimal response (minimal viability, i.e., 0 when cell viability is 0%, or unconstrained when dose–responses do not achieve 100% cytotoxicity), Y = 50 (i.e., 50% response), and HillSlope expresses the steepness of the curve. The determination coefficient R^2^ was used for the evaluation of the capability of the rearranged Hill function to quantitatively interpret the experimental data.

The rearranged Hill function offers the capability to evaluate the slope of the dose–response curve, which is extremely important for the prediction of the toxicity of a substance at certain doses, and reflects the speed of increase in response as the concentration rises. IC_50_ data are shown as the mean ± SD of at least two independent experiments, which were carried out in triplicate. A linear regression analysis was also performed using the corresponding LD_50_ values provided by the ICCVAM [5], and the r^2^ coefficient was used for quantitative evaluation of the performed regression analyses. The obtained regression was then compared to those of HepG2 and NIH 3T3 cells through F-tests. The obtained IC_50_ data were also used to predict corresponding LD_50_ values and GHS hazard categories using the RC rat-only millimole regression: log LD_50_ (mmol/kg) = 0.439 log IC_50_ (mM) + 0.621 (applicable to substances of known molecular weight); and the RC rat-only weight regression: log LD_50_ (mg/kg) = 0.372 log IC_50_ (ug/mL) + 2.024 (for mixtures or other substances of unknown molecular weight), as recommended by the ICCVAM [5,6].

The precision, heterogeneity, and reproducibility of our WJSC-based assay was evaluated through determining (a) intra-assay variation (i.e., the % coefficient of variation (CV) within each microtiter plate (MTP) or for each chemical), (b) inter-assay variation (%CV between different MTPs, or corresponding to the IC_50_ of different chemicals,) (c) inter-culture variation (difference in slopes and intercepts of linear regressions of WJSCs derived from different tissue samples), and (d) by comparison of inter-laboratory variation (CV%) of the IC_50_ values calculated based on the standard NHK-NRU toxicity testing of the full panel of 12 chemicals, as reported by three different ICCVAM laboratories [5], to that of WJSC-NRU in our study.

### 2.10. Cluster Analysis

The hierarchical agglomerative cluster analysis conducted on lethal dose data included human minimum lethal dose (LD_Lo_; according to MEIC study [22,23]), rat LD_50_, per os (according to RC [9]), as well as LD_50_ values that were derived from the respective in vitro IC_50_ values by conversion via the RC rat-only weight equation. Analysis was conducted separately on two sets of data: one corresponding to 12 chemicals, and one to a subset of 7. Scaling of observed values was applied, while two clustering methods were employed—average linkage and Ward’s criterion—generating similar results. Cluster analysis was carried out by using the Multibase program as an add-on in Excel.

## 3. Results

In the present study, we estimated the ability of our WJSC-based assay to correctly predict both the hazard category and the in vivo acute oral toxicity levels of 12 chemicals by means of two types of regressions. Two different colorimetric methods were employed in order to determine cell viability/toxicity endpoints (MTS and NRU assays). A list of properties of the selected chemicals is shown in Table 1.

The toxicity prediction scores of the WJSC-based model were compared to those provided by another primary MSC type—ADSCs—and also NIH 3T3 and HepG2 cells. Moreover, we compared the results on WJSCs with those of the ICCVAM-validated cell lines BALB/c 3T3 and NHK, as well as with BMSCs (in silico results). Toxicity screening also took place in 3D cultures of WJSCs, which are believed to better reflect the actual cell growth environment that occurs within the body. Finally, we evaluated the precision, heterogeneity, and reproducibility of our WJSC-based assay, as described above. The experimental overview is presented in Table 2.

In terms of prediction of the correct hazard classification of the tested chemicals, when the NRU assay was used, both the RC rat-only millimole and RC rat-only weight regressions correctly predicted the GHS category for 41.7% (5/12) of the tested chemicals; in vivo toxicity was underpredicted by 41.7% (Table 3). For the WJSC-MTS test, the use of both regressions provided correct prediction in 41.7% of the compounds tested. The frequency of underprediction was again, as in the case of NRU, higher than that of overprediction (5/12 vs. 2/12).

**Figure 1:**
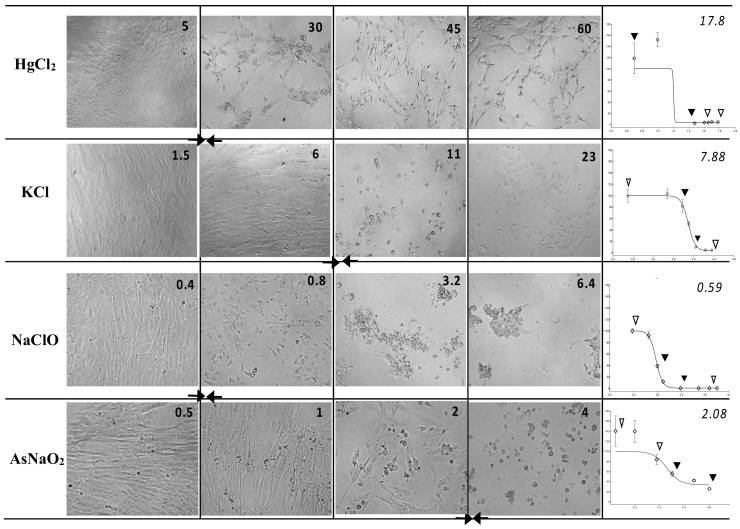
Cytotoxicity in WJSCs following 48 h of exposure to various concentrations of chemicals—Left panel: micrographs (25×) of WJSCs 48 h after exposure to four chemicals representative of four hazard classes; the four concentrations are shown from highest (far right) to lowest (leftmost column). Figure inserts in top right corner of each micrograph denote the exact concentration (in ug/mL) of the chemical used. Right panel: respective survival curves (y = % viability, x = concentration in log ug/mL) for each chemical and their IC_50_ values (ug/mL; top right corner of plots). Arrows on survival curves depict the four respective concentrations shown on the micrographs on the left. Diamonds depict all tested concentrations. IC_50_ values correspond to the curve area between the two solid arrowheads and the culture status delineated by the micrographs marked by two solid arrows in the left panel.

The WJSC screening test provided correct toxicity class prediction, as verified by both types of RC rat-only regression analysis, for three chemicals—KCl, propranolol hydrochloride, and glycerol—irrespective of the evaluation method (MTS or NRU). Moreover, the WJSC-NRU and WJSC-MTS tests correctly predicted the GHS category for two (sodium dichromate dihydrate and atropine sulfate monohydrate) and three (sodium fluoride, cadmium(II) chloride, and atropine sulfate monohydrate) additional drugs, respectively. In the case of incorrect estimation of toxicity, overprediction was less frequent than underprediction for both NRU and MTS, and was mainly observed for less toxic grade 6 chemicals (Table 3 and Figure S1). It is worth noting that based on the regression values of Table 3, the NRU assay shows a tendency to be relatively more accurate (values closer to LD_50_) for highly toxic chemicals, while on the other hand, MTS gives better prediction scores for low-toxicity drugs. Representative images of cytotoxicity in WJSCs following 48 h of exposure to various concentrations of chemicals, along with the respective IC_50_ values, are presented in Figure 1.

The predicted LD_50_ scores for two inorganic chemicals of moderate-to-high toxicity—CdCl and NaF—were found to be borderline correct and borderline underpredicted, respectively, as determined by the RC rat-only weight regression and the WJSC-MTS assay (Table 3). We hypothesized that the correct determination of the IC_50_ and, consequently, of predicted LD_50_ was hindered by neutralization of cytotoxicity by serum components. With this in mind, we determined the IC_50_ values for these two chemicals by conducting the WJSC-MTS assay using the same growth medium (DMEM/F12), but without the addition of FBS. Serum-free conditions resulted in lower predicted LD_50_ values, closer to the in vivo LD_50_ data, and actually enabled the correct prediction of NaF toxicity, improving the overall correct prediction rate of the weight regression to 50% (6/12), preceding the respective rate of the millimole regression (Table 3). It is worth noting that the viability for WJSC cultures maintained in serum-free conditions, and in the same timeframe used for toxicity testing, remained consistently high (>80%) compared to cells grown in normal GM with 10% FBS.

**Figure 2:**
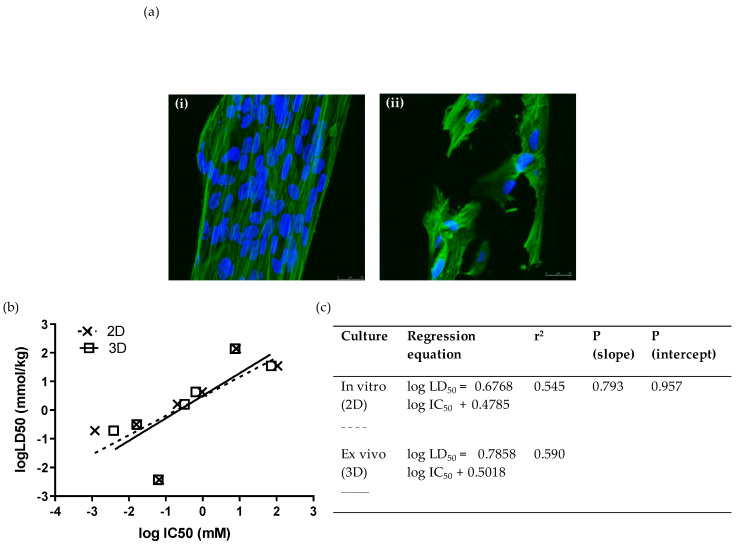
Ex vivo cytotoxicity screening of 7 selected chemicals on 3D scaffolds by means of the WJSC-MTS assay: (**a**) Growth and morphology of WJSCs in the 3D microenvironment of the inert polystyrene (PS) scaffold. Panels show representative composite confocal micrographs of WJSCs growing on the periphery of the microtubules comprising the scaffold meshwork (**i**) in the presence of growth medium (GM) only (control), or (**ii**) in GM containing a concentration of NaF close to its IC_50_. Each composite image was derived by merging 10 confocal micrographs taken across the tube diameter at 1.5 um steps (z-series). Mag = 40×, Mag bar = 25 uM; blue = DAPI nuclear staining; green = phalloidin staining of the actin cytoskeleton. (**b**,**c**) Comparison of linear regressions of human fetal WJSCs cultured on conventional tissue-culture-treated plastic (polystyrene (PS)) surface (2D culture), and in three-dimensional inert scaffolds with a rectangular mesh structure (PS inserts).

In addition to screening in standard two-dimensional cultures of WJSCs, we also conducted analyses in a 3D ex vivo culture format using inert polystyrene scaffolds, which are believed to provide a more representative environment of in vivo pharmacodynamics. Moreover, since the scaffold was made from the same material as a standard in vitro culture, any difference in the assay performance characteristics would be essentially attributed to the difference in the spatial/architectural organization between the two culture systems. We tested the toxicity (by means of MTS assay) of seven compounds (at least one from each hazard category) on PS 96-well disc insets seeded with WJSCs after 48 h. WJSCs were fixed and stained with DAPI and phalloidin for the visualization of the nucleus and cytoskeleton, respectively (Figure 2a). The IC_50_ values obtained showed excellent correlation with 2D culture (Figure 2b,c). Comparison of the linear regressions for toxicity estimation in 2D and 3D cultures showed that they did not differ statistically (*p* (slope), *p* (intercept) > 0.05). Moreover, the prediction rate was the same as for the respective 2D cultures when the RC rat-only millimole regression was used (Table 3).

**Figure 3:**
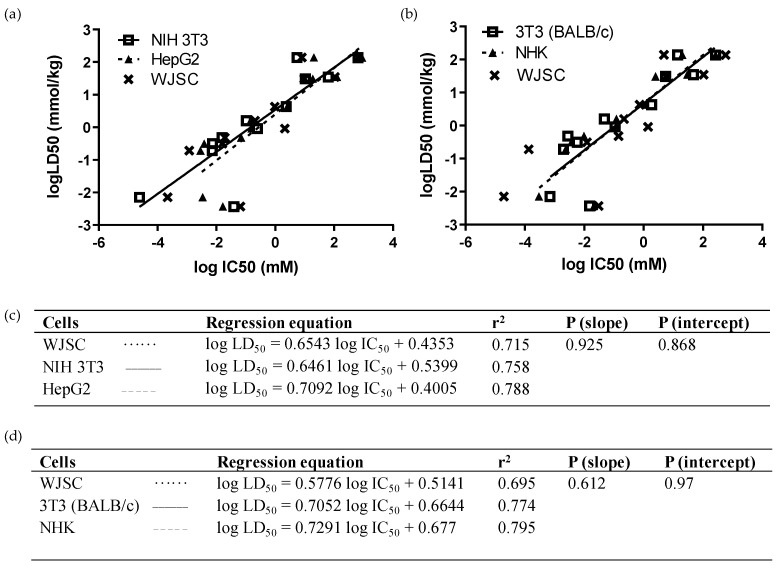
Comparison of linear regressions of WJSCs with human and murine cell lines after 48 h of treatment with the 12 selected chemicals: (**a**,**c**) In vitro comparison with ΝΙH 3Τ3 (solid line, open squares) and HepG2 cells (dashed line, closed triangles). Data were obtained by means of the MTS assay. Regression equations, r^2^, and *p*-values were generated and used to compare predictability of the assays. (**b**,**d**) In silico comparison with BALB/c 3Τ3 (solid line, open squares) and NHK cells (dashed line, closed triangles). Data for WJSCs were obtained via the NRU method in our lab, while data for BALB/c 3T3 and NHK cells, and the respective LD_50_ values, were derived from the ICCVAM validation study [6]. The WJSC regression line is represented in both analyses by a dotted line with data points marked as “x” symbols. Regression equation, r^2^, and *p*-values were generated and used to compare the predictability of the assays.

In turn, we performed multiple comparisons regarding the predictability of compound toxicity between our WJSC-based model and other validated cell lines or adult MSC types. As depicted in Figure 3, linear regressions of LD_50_ values, extracted from MTS viability results using the IC_50_ values of the 12 chemicals, did not differ significantly between WJSC, NIH 3T3, and HepG2 cells (*p* (slope), *p* (intercept) > 0.05; Figure 3a,c). The r^2^ values, depicting goodness of fit, were also similar (Figure 3c), while the gap in r^2^ was further shrunk when WJSCs were cultured in serum-free conditions (Table 4). However, when the NRU assay was used to compare linear regressions for the 12 chemicals between WJSCs and the in silico data of the two ICCVAM-validated cell lines, there was a slight deviation between WJSCs and BALB/c 3T3 and NHK cells, represented by differences in r^2^ values, though linear regressions did not differ statistically (*p* (slope), *p* (intercept) > 0.05; Figure 3b,d). The correct toxicity prediction rate of the 12 chemicals was equal or higher for WJSC-MTS (41.7/50%) and for WJSC-NRU (41.7%), as compared to each one of the above cell types, regardless of the regression type (weight vs. millimole)—except for HepG2, which gave a high prediction rate (67%) when the RC rat-only millimole regression was used (Table 4), which can mostly be attributed to the higher sensitivity of these cells against chemicals with liver-specific toxicity, such as sodium arsenite.

**Figure 4:**
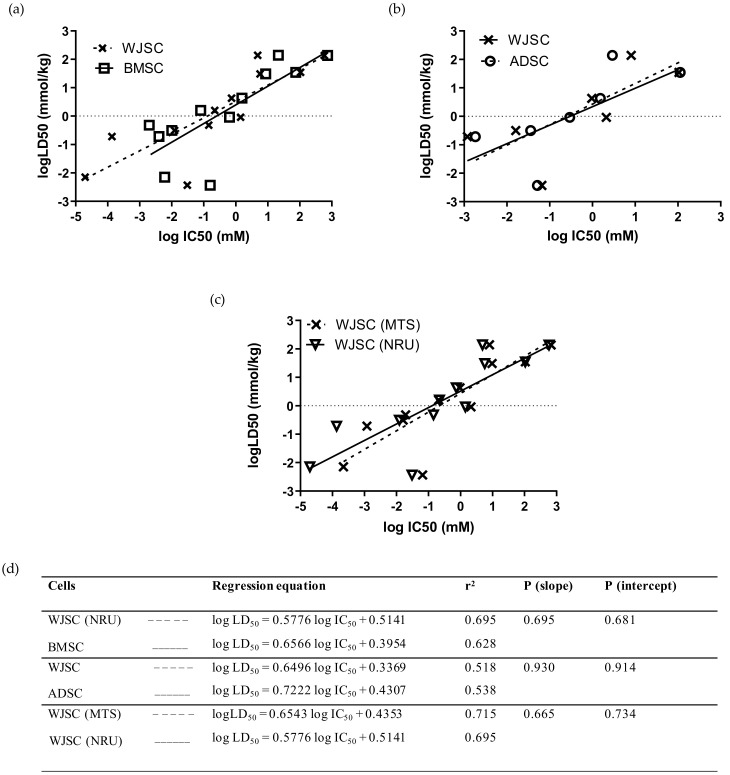
Comparison of linear regressions of human fetal WJSCs with two types of human adult MSCs: (**a**) Comparison with BMSCs for the full panel of 12 chemicals using the NRU assay. (**b**) Comparison with ADSCs for a subgroup of seven selected chemicals (mercury(II) chloride, sodium arsenite, sodium dichromate dihydrate, sodium fluoride, propranolol hydrochloride, potassium chloride, and sodium hypochlorite) by means of MTS assay. (**c**) Comparison of the linear regressions generated by evaluating the toxicity of 12 chemicals on WJSCs using the MTS and NRU assays. The regression data presented here were generated by independent in vitro experiments in our lab, except for data regarding BMSCs and LD_50_ values, which were derived from the work by Scanu et al. [14] and by the ICCVAM validation study [6], respectively. Linear regression plots (top panel) are shown as dashed lines with data points marked as “x” symbols (WJSCs), solid lines with open squares (BMSCs), and dashed lines with open circles (ADSCs). (**d**) Regression parameters in tabular format for each of the regression graphs. In all cases, regressions did not differ significantly (*p* > 0.05, F-test).

Comparison of linear regressions for the full panel of 12 chemicals between MSCs of fetal and adult origin showed that the data based on the WJSC-NRU assay had a slightly higher r^2^ coefficient than the data derived from BMSCs, although linear regressions did not differ statistically (*p* (slope), *p* (intercept) > 0.05; Figure 4a,d), and acute oral toxicity prediction rates were identical (41.7%) for the two stem-cell-based models (Table 4). Similarly, comparison of linear regressions for the seven selected chemicals—at least one per hazard category (mercury(II) chloride, sodium arsenite, sodium dichromate dihydrate, sodium fluoride, propranolol hydrochloride, potassium chloride, and sodium hypochlorite)—between WJSCs and ADSCs resulted in regression equations with similar r^2^ coefficients and statistically similar linear regressions (*p* (slope), *p* (intercept) > 0.05; Figure 4b,d). However, the correct toxicity prediction rate was higher for WJSC-NRU (42.9%) compared to ADSC-NRU (14.3%) when the RC rat-only millimole regression was used (Table 4). Direct comparison between the two selected assays resulted in similar regression coefficients and linear regressions, with no statistically significant differences (*p* (slope), *p* (intercept) > 0.05; Figure 4c,d); meanwhile, regarding efficacy, the WJSC-MTS assay gave similar toxicity prediction scores to the WJSC-NRU (Table 4). Comparison of the experimentally and theoretically (using the RC rat-only weight regression equation) determined IC_50_ values between WJSCs and the other cell types employed in this study is shown in Figure S1.

**Figure 5:**
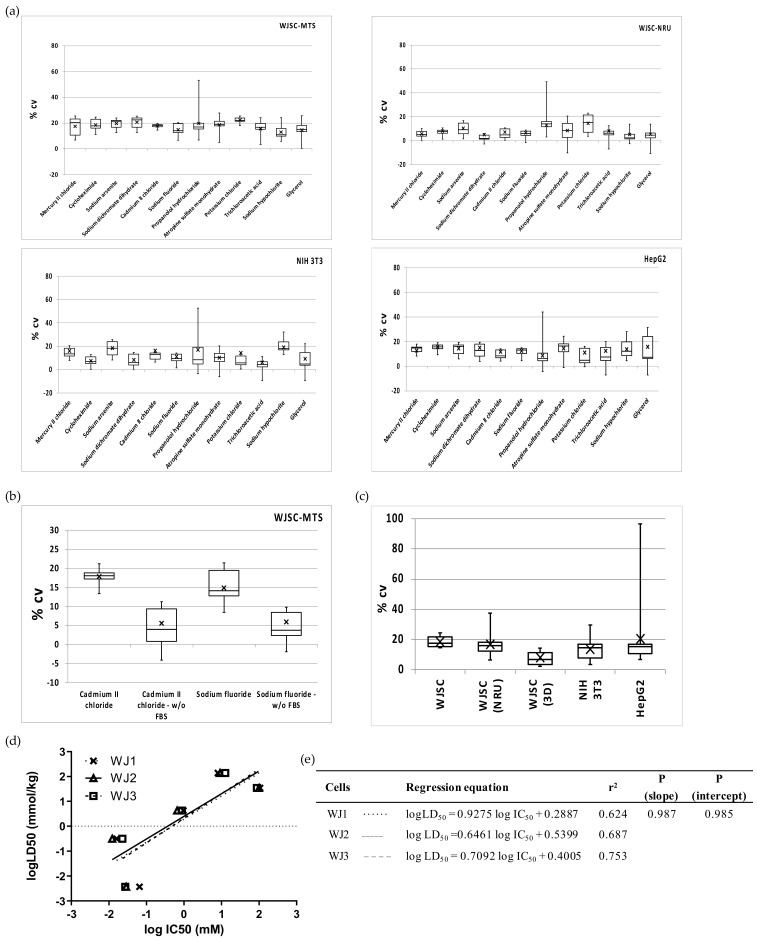
Precision of the WJSC-based toxicity assay: (**a**) Intra-assay variations (%CV within each MTP/for each chemical) of 2D culture assays for WJSC, NIH 3T3 and HepG2 cells. Chemicals are listed on the x axis in descending order of cytotoxicity. Cell viability/toxicity in WJSCs was estimated using either the MTS (upper left) or the NRU (upper right) assays, while in both NIH 3T3 and HepG2 cells the MTS assay was employed (down). (**b**) Effect of FBS on intra-assay variation of the WJSC-MTS assay; two chemicals were used—cadmium(II) chloride and sodium fluoride, *p* < 0.001 standard culture vs. no FBS, for both chemicals. (**c**) Inter-assay variation calculated at IC_50_ (*n* = 12 in all cases, apart from 3D culture, where *n* = 7). (**d**) Inter-culture variation in the WJSC cytotoxicity screening assay. The linear regressions for five selected chemicals (mercury(II) chloride, sodium arsenite, sodium fluoride, potassium chloride, and sodium hypochlorite) are shown in comparison of human fetal WJSCs derived from three different donors (WJSC #1, WJSC #2, WJSC #3). Regression lines are plotted as a dotted line with data points marked as “x” symbols, a solid line with open triangles, and a dashed line with open squares for WJSC #1, WJSC #2, and WJSC #3, respectively. (**e**) Regression parameters in tabular format for each of the regression graphs. The three regressions did not differ significantly (*p* > 0.05, F-test).

The heterogeneity/precision of the WJSC screening assay was evaluated by measuring (a) the intra-assay variation (%CV within each MTP/for each chemical), (b) the inter-assay variation (%CV between different MTP/corresponding to IC_50_ of different chemicals), and (c) the inter-culture variation (differences in the slope and intercepts of linear regressions of WJSCs derived from different tissue samples). Intra-assay variations calculated for each chemical did not differ significantly, and this was true for all three cell types (ANOVA, *p* > 0.05; mean %CV +/− SD = 17.7 ± 2.8, 12.9 ± 4.5 and 13.2 ± 2.1, for WJSCs, NIH 3T3, and HepG2, respectively; Figure 5a), highlighting that the toxicity of each chemical is not related to variation. However, value spread/dispersion (see boxplots in Figure 5a) was overall lower for WJSCs (interquartile range (IQR) of 5.5 vs. 7.9 for NIH 3T3 and 8.1 for HepG2). Nevertheless, it is worth noting that propranolol hydrochloride generated intra-assay variation values with the highest dispersion in all of the tested cell lines (Figure 5a). With respect to the detection method that was employed, the NRU assay generated significantly lower intra-assay variation for all chemicals, with the exception of propranolol hydrochloride and KCl. Nevertheless, inter-assay variation ranged between 13% and 20%, and did not differ significantly between the groups (Figure 5c). Interestingly, when we evaluated toxicity in serum-free conditions (for two selected chemicals with borderline prediction scores, as discussed above), the variance in WJSCs was significantly diminished (*p* < 0.001) by almost threefold. The variation between distinct cultures of WJSCs derived from three different donors (used at passage 2 for WJSC #1 and WJSC #2, which were isolated from the umbilical cords of heterozygotic twins, and at passage 6 for WJSC #3) showed that the sample-related heterogeneity (biological/culture variation) is not greater than the technical/assay variation (Figure 5d,e).

**Figure 6:**
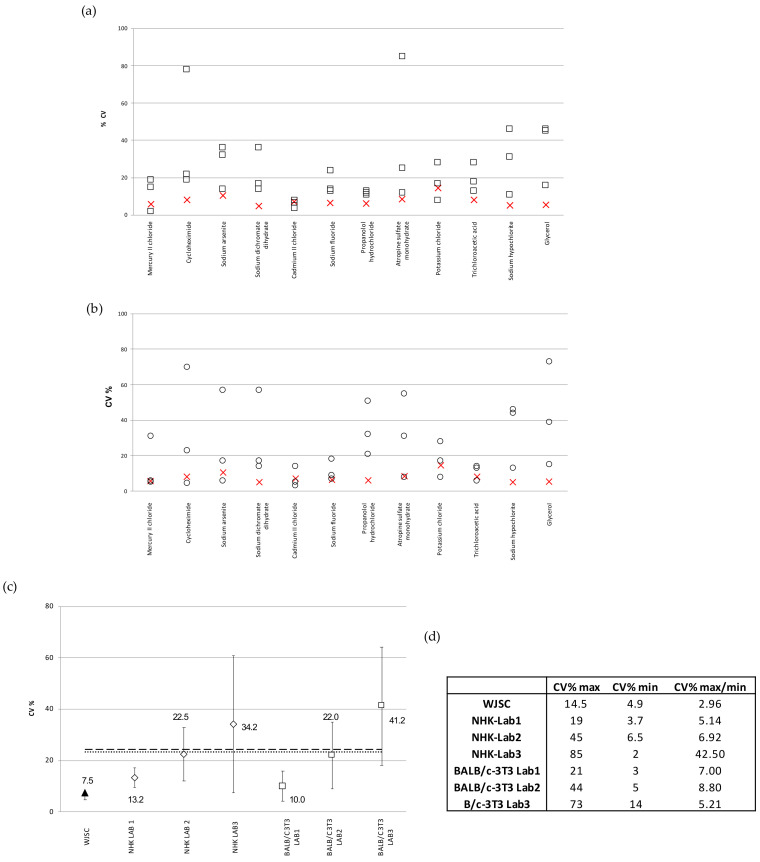
Reproducibility of the WJSC-based toxicity assay: (**a**) Comparison of inter-laboratory variation (CV%) of IC_50_ values calculated by NHK-NRU toxicity assay in three different laboratories selected by the ICCVAM [6] (values displayed as squares), with those calculated based on the WJSC-NRU assay in our laboratory (values shown as “X”). The three plus one values in each column are the CV% values as recorded in the three ICVAAM laboratories and in our laboratory, respectively, for each of the 12 different substances, presented in descending order of toxicity from left to right along the x axis; (**b**) Comparison of inter-laboratory variation (CV%) of IC_50_ values calculated based on the BALB/c 3T3-NRU toxicity assay (in three different laboratories selected by ICCVAM; values are shown as squares) with those calculated based on the WJSC-NRU assay in our laboratory (values shown as “X”). The three plus one values in each column are the CV% values as recorded in the three ICVAAM laboratories and in our laboratory, respectively, for each of the 12 different substances, presented in descending order of toxicity from left to right along the x axis. (**c**) Comparison of the mean inter-laboratory variation (CV% ± standard deviation) of IC_50_ values derived from the three experimental triplicates with the lowest deviation for the total of 12 substances; horizontal lines = mean CV% of three labs (dashed = 3T3, dotted = NHK. (**d**) Mean inter-laboratory variation values (CV%) for the total of 12 substances tested.

The reproducibility of the results given via the WJSC cytotoxicity assays was also evaluated in relation to the NHK and BALB/c 3T3-NRU assays performed in ICCVAM-selected laboratories, as described in [5]. Figure 6a depicts the inter-laboratory variability (CV%) of the IC_50_ values calculated based on the standard NHK-NRU toxicity testing of the full panel of 12 chemicals in 3 different ICCVAM laboratories, in comparison to the WJSC-NRU assay (*n* = 4). WJSC-NRU had the lowest variability for 9 out of the 12 substances tested, with CV% values ranging from 4.9 to 14.5 (inter-laboratory variation = 7.5%). This variation was the lowest compared to those reported by the three ICCVAM labs, which averaged 23.3%. Similarly, in comparison to the BALB/c 3T3-NRU, the WJSC-NRU assay had the lowest variability for 7 out of the 12 substances; the inter-lab variations of the three ICCVAM labs in this case ranged from 3 to 73%, which again were much higher (over threefold on average) compared to that of WJSC-NRU (Figure 6b). Examination of the data referring to those three experimental triplicates with the lowest mean inter-laboratory variations in IC_50_ values (CV% ± standard deviation) between laboratories showed that the WJSC-NRU test has the lowest variability, with CV% being at least three times lower than the respective average CV% of the other three laboratories (Figure 6c). Overall, the WSJC-NRU assay exhibited the lowest inter-laboratory variation compared to both 3T3- and NHK-NRU assays, as evidenced by the low (<3) maximum–minimum value of inter-laboratory CV% (Figure 6d).

**Figure 7:**
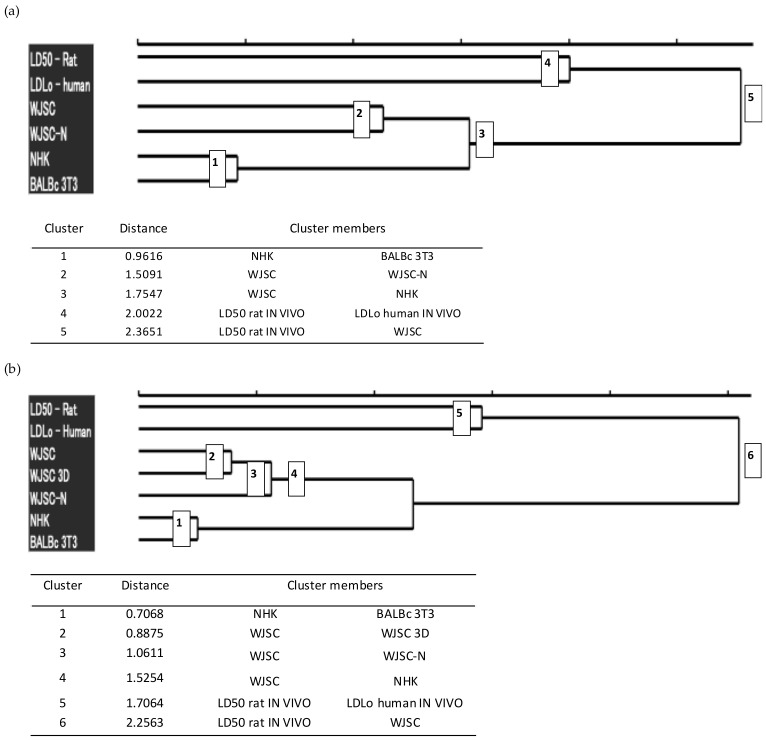
Hierarchical agglomerative cluster analysis of lethal dose data: Dendrograms were constructed using the lethal dose values (human LD_Lo_, rat per os LD_50_, and LD_50_ derived from IC_50_ values of cell cultures). (**a**) The upper dendrogram shows clustering of lethal doses for the full panel of 12 chemicals studied, while the lower table includes additional data, such as the number and composition of the clusters, and their degree of dissimilarity (Euclidean distance between cluster members). The analysis revealed five main groups that are shown in the dendrogram as nodes whose number corresponds to the order of classification on ascending distance. (**b**) The upper dendrogram shows clustering of lethal doses for a restricted subset of LD_50_ data that correspond to the 7 chemicals for which toxicity was also evaluated via the MTS assay in 2D and 3D cultures for WJSCs, while the lower table includes additional data, such as the number and composition of the clusters, and their degree of dissimilarity. The analysis revealed six main groups that are shown in the dendrogram as nodes whose number corresponds to the order of classification on ascending distance. The analysis was performed on scaled data using both average linkage and Ward’s clustering methods, which produced similar results. WJSC: measured by the MTS assay; WJSC-N: measured by the NRU assay. Data for NHK and BALB/c were retrieved from the ICCVAM report [5].

For further investigation of the quality of the results obtained from the cytotoxicity assays, we performed hierarchical agglomerative cluster analysis of these results into groups, based on the available/extracted lethal dose data (Figure 7). Cluster analysis was performed using the human minimum lethal doses (lethal dose low, LD_Lo_) (according to the MEIC study [22]), LD_50_ values in rats (oral administration according to RC [22]), and LD_50_ values derived from the corresponding IC_50_ values via conversions based on RC rat-only weight equations.

Based on the results, the LD_50_ values that were calculated from the WJSC-based assays show a greater similarity to the actual toxicity values that have been established for humans (LD_Lo_) and rats (LD_50_) than those resulting from the validated cell models for cytotoxicity assessment (NHK-NRU, BALB/c-NRU). In addition, the LD_50_ values resulting from the application of the WJSC-MTS assay are the only ones that form a cluster with the in vivo values (LD_50_), thus highlighting a higher correlation between these values. Taken together, these data demonstrate the superiority of the WJSC-based assays and their suitability as an effective alternative to animal toxicity testing.

## 4. Discussion

It is widely recognized that in vitro basal cytotoxicity test methods, as part of a weight-of-evidence approach to estimate the starting doses for acute oral in vivo toxicity test methods, should be considered and used where appropriate before testing is conducted using animals. Although the BALB/c 3T3- and NHK-NRU assays constitute validated, widely adopted toxicity testing methods, they utilize highly differentiated cells that are unsuitable for providing the best prediction of acute lethality for the large variety of chemicals likely to be tested for acute toxicity [22,23]. Stem-cell-based toxicity screening assays offer an attractive alternative to established drug screening methodologies that rely on animal experimentation.

In the present study, we sought to evaluate the applicability of human WJSCs as a cell model for an in vitro cytotoxicity test that could correctly predict LD_50_ and the hazard category according to the GHS [8]. We adopted a 96-well plate high-throughput screening (HTS) platform on which WJSCs’ viability after 48 h of exposure to a range of concentrations of each selected chemical was measured by means of the MTS viability assay to determine the IC_50_. A second end-point viability assay—the more widely used NRU assay—was also used for comparison. Our results show that the human WJSCs match the ICCVAM requirements for accuracy and reliability, since the WJSC regressions obtained were not statistically different from those related to the two already validated BALB/c 3T3 and NHK cell lines, based on the comparison of the slope and intercept. Moreover, WJSC-MTS/-NRU assays were able to predict toxicity with comparable accuracy to both reference cell lines. However, the WJSC-MTS/-NRU assay is able to correctly predict the toxicity of slightly toxic chemicals, such as glycerol, regardless of the regression model employed; in contrast, both the BALB/c 3T3 and NHK validated methods overpredicted the toxicity of both GHS class 6 chemicals tested under both regression models. Although characterized by a similar ability to correctly predict toxicity, WJSC-based assays offer significant advantages over the use of other cell types.

Several studies, such as the MEIC study, have shown that almost any cell type could be used for the measurement of basal cytotoxicity [22,23,24]; however, human cell lines are more suitable for detecting cytotoxicity than cells of animal origin. A long-term advantage of using human cells is that cytotoxicity results can be added to human toxicity databases to facilitate the development of methods to predict acute human lethality, including valuation of the contribution of genetic background variations to susceptibility to toxicity. Our multiple comparisons indeed verify the general observations of the MEIC study, with all cell lines exhibiting a basal toxicity response to the 12 chemicals tested. HepG2 cells gave the best predictability of all the tested cell lines; however, several studies have highlighted the poor biological representation of human primary hepatocytes by this transformed cell line [10,25]. With respect to stem cells, ADSCs have shown great potential for use in cytotoxicity studies [26,27], but the biological properties of in vitro expanded populations—including slow expansion rate and low yield—render their use considerably cumbersome. With an average PDT of over 400 h, ADSCs fail to meet the ICCVAM criterion of a maximum PDT of 36 h. This, coupled with increased heterogeneity with respect to their isolation and during subculture, renders them unsuitable for high-throughput screening. The rapid expansion potential (average PDT of 28.8 h [18]) of WJSCs allows for the derivation of at least 100 million cells from a single sample, which can be used to screen for over 550 chemicals according to our protocol. Accordingly, this provides the capability to produce large numbers of cells for extensive toxicity testing without the need for continuous isolation of cells from tissues, thus avoiding the risk of biological and technical heterogeneity.

With respect to assay reproducibility, WJSCs showed excellent homogeneity and uniformity. Equally importantly, the low variation observed between cultures of WJSCs derived from different donors signifies that sample-related heterogeneity (biological/culture variation) is not greater than technical/assay variation, which is usually the case for primary cells. In total, both intra- and inter-assay variations for WJSC-based assays were lower than the mean intra-laboratory CV% reported for NHK- and BALB/c 3T3-NRU, while these can be further improved by depletion of FBS from the culture medium.

Serum interference has been recognized as a cause of toxicity underprediction in many drug and other compound cases [28,29,30,31]. In our hands, growth of WJSCs in serum-free standard medium for the last 48 h of cell culture resulted in a 30% decrease in the calculated mg/kg values for class 3 chemicals, reducing underestimation of their toxicity. With respect to validated assays, it is worth noting that one of their key differences concerns serum requirements. Thus, whereas murine 3T3 cells grow in standard FBS-containing medium, maintenance of human primary NHKs requires the use of chemically defined media, rendering their culture more cumbersome and considerably (up to 5 times) more expensive; considering this cost alone, the ICCVAM suggests the use of BALB/c 3T3 cells, since they yield comparable results. In this context, the WJSC-based acute toxicity assay represents an attractive alternative to NHK-NRU assay, combining lower operating cost and reduced variability with similar or better toxicity prediction rates. Moreover, serum withdrawal ameliorates all three aspects of assay performance, most notably improving the correct toxicity class prediction score by 25%.

With respect to the end-point assay, we determined cytotoxicity/viability levels mainly by means of the MTS assay, but also with the popular, validated neutral red uptake (NRU) assay. Both assays determine endpoint cell viability, albeit based on the expression of different markers, i.e., metabolic (mitochondrial dehydrogenases) and membrane markers (lysosomal storage) in the case of MTS and NRU assays, respectively. The MTS assay belongs to the same group of colorimetric assays as MTT, XTT, and WST-1; however, it has been shown to be superior to other tetrazolium salt assay variants in terms of accuracy, reliability, and ease of application [32,33,34]. In comparison to NRU, in our experiments there was an excellent correlation with MTS, with no significant difference in assay variation. Moreover, although the NRU endpoint is suitable for certain tissue-specific in vitro assays, its universal suitability for cell-based assays is questionable. Certain drugs and chemicals, such as chloroquine and specific surfactants, have been shown to locally interact with lysosomes, thus leading to anomalous results [35,36]. Technical issues such as dye precipitation into crystals and the development of circular areas of cell death within the MTP can also hinder its accuracy. In any case, more data stemming from the comparison of MTS versus NRU are needed. Unquestionably, the choice of end-point assay adopted for in vitro toxicity studies is crucial [37]. On this note, cluster analysis of our data highlighted the WJSC-MTS assay as the only assay that clustered with in vivo assays.

We adapted our protocol to include toxicity screening in a 3D environment, in an effort to test the hypothesis that the spatial culture environment plays a crucial role in cell attachment and growth, and ultimately affects cells’ sensitivity to drugs. Several reports suggest that 3D culture allows for the development of pharmacodynamics that more closely resemble the in vivo situation [38,39,40]. Our results clearly show that WJSCs can adhere and grow well on a 3D culture substrate. In response to toxic insult, this attachment/growth pattern is disrupted, and cells detach into the surrounding space. In the subset of seven chemicals that we evaluated, 3D culture did not improve the prediction score, but gave slightly higher correlation coefficients compared to all other assays employing WJSCs, while it also gave CV% values that were 2.5-fold lower than in 2D culture. The results presented here look promising, and form a sound basis for a more thorough optimization of ex vivo drug screening. Ultimately, we envisage that the co-establishment of 3D culture setup with directed differentiation protocols will aid in the development of organotypic cultures suitable for testing organ-specific toxicity, expanding the use of WJSCs beyond basal toxicity evaluation.

Overall, although an effort was made to adopt a comprehensive experimental design that would allow multiple aspects of the toxicity screening competence of our WJSC-based model to be examined, our study leaves a few issues to be addressed. For example, extreme toxicity (class 1 and 2 chemicals) is still underpredicted; this inherent inadequacy of in vitro cultures to mimic the kinetics and dynamics of substances related to an in vivo system stems from their lack of absorption, distribution, metabolism, and excretion (ADME) mechanisms, which normally control the exposure of the target tissues to the toxicants in vivo. Expanding the studies to include other chemicals that exhibit both organ-specific and general toxicity, along with the inclusion of biokinetic data, might improve the correlation/prediction rates of the WJSC-based model. It would also be interesting to directly correlate IC_50_ with LD_Lo_ in humans (MEIC), which was beyond the scope of this study.

## 5. Conclusions

Overall, in this study, we present for the first time evidence in support of the feasibility of use of MSCs isolated from human fetal tissue for acute toxicity screening. Our data suggest that WJSC-based cytotoxicity assays perform at least as well as validated in vitro assays, with comparable toxicity hazard predictability as well as reproducibility. Moreover, adoption of WJSCs alleviates two of the main drawbacks of these assays, namely, the questionable translation, due to interspecies differences, of BALB/c 3T3 data to humans, and the higher costs associated with manipulation of NHKs in vitro. It is also worth noting that WJSC multipotency enables the potential evolution of assay functionality, from basal toxicity evaluation to organ-specific and developmental toxicity screening. In any case, wide adoption and cross-validation of the assay to cover a broader range of chemicals, chemical mixtures, and different endpoints and culture settings would generate enough information to facilitate the transformation of toxicology from assessment of apical endpoints in animals to mechanistically relevant studies that primarily rely on the combination of in vitro assays and computational (in silico) methods based on human biology, resulting in large-scale savings in time, costs, and animals, in full concordance with the “3 Rs” principle put forward over half a century ago.

## Figures and Tables

**Table 1:** Properties of the 12 chemicals used in the present study.

Chemical (CAS-Nr)	Formula	Mw	Ontology/ Properties	Usage	Route of Metabolism/ Mode of Action/ Target Organ	Hazard Class (LD_50_, mg/kg)
Sodium dodecyl sulfate (SDS)	C_12_H_25_NaO_4_S	288.38	Comp. (org), Anionic surfactant, amphiphilic	Detergent, SDS–PAGE		(+) control
Mercury(II) chloride	HgCl_2_	271.5	Subst. (in), Corrosive	Antiseptic/disinfectant	Kidney	1 (≤5)
Cycloheximide	C_15_H_23_NO_4_	281.39	Comp. (org)	Antibiotic (*Streptomyces*/antifungal)	Inhibition of eukaryotic protein synthesis/liver	1
Sodium arsenite	AsNaO_2_	129.91	Subst. (in), carcinogenic	Pesticide (rat poison)	Enzyme inhibitor/liver, skin	2 (>5–50)
Sodium dichromate dihydrate	Na_2_Cr_2_O_7_·H_2_O	298	Comp. (org), oxidizing agent Cr(VI), highly reactive/corrosive, genotoxic carcinogen	Leather tanning	Lungs, liver, skin	2
Cadmium(II) chloride	CdCl_2_	183.32	Subst. (in), hygroscopic, corrosive	Photograph and fabric printing, electroplating, preparation of Cd yellow pigment	Kidney, liver	3 (>50–300)
Sodium fluoride	NaF	41.99	Subst. (in)	Dentistry, water treatment, fluorocarbon synthesis, PET imaging	GI irritant, CNS depressant	3
Propranolol hydrochloride	C_16_H_21_NO_2_	295.8	Comp. (org), sympatholytic, lipophilic	Treatment of hypertension, anxiety, panic, glaucoma, tremor	Non-selective beta blocker	4 (300–2000)
Atropine sulfate monohydrate	(C_17_H_23_NO_3_)_2_ H_2_SO_4_·H_2_O	694.83	Comp. (org), anticholinergic (parasympatholytic)	“Essential drug” (WHO), resuscitation), mydriatic (ophthalmology), sludge treatment (organophosphate poisoning)	Competitive antagonist of muscarinic acetylcholine receptors	4
Potassium chloride	KCl	74.56	Subst. (in)	Fertilizer (potash)	Cardiotoxin	5 (2000–5000)
Trichloroacetic acid	C_2_HCl_3_O_2_	163.39	Caustic acid	DNA/RNA/protein precipitation, cosmetics (skin peeling)	GI corrosion, acidosis	5
Sodium hypochlorite	NaClO	74.44	Comp. (in), oxidant, corrosive	Disinfectant, bleaching agent, water treatment	Body fluids	6 (>5000)
Glycerol	C_3_H_8_O_3_	92.09	Comp. (org), hygroscopic	Humectant, solvent, sweetener, food additive, soap-making	Osmosis/body fluids	6

Comp. = compound; Subst. = substance; In = inorganic; Org = organic; GI = gastrointestinal tract; CNS = central nervous system; PET = positron emission tomography; WHO = World Health Organization.

**Table 2:** Tabular overview of the experimental design employed for the evaluation of the WJSC-based acute toxicity assay.

Evaluation Parameter	Assessment Method	WJSC-Based Acute Toxicity Assay		No. of Chemicals Tested	Cross-Validation Cellular Assays/Data		Data Source Type *	
Toxicity prediction capacity	Comparison of linear regressions	WJSC	MTS	12	NIH 3T3	MTS		Human cell lines
					HepG2	MTS		
		WJSC	NRU	12	Balb/c-3T3	NRU	In Silico	ICCVAM VALIDATED
					NHK	NRU	In Silico	
		WJSC	MTS	7	ADSC	MTS		MSC (human adult)
		WJSC	NRU	12	BMSC	NRU	In Silico	
		WJSC	MTS	12	WJSC	NRU		MSC (human fetal)
		WJSC	MTS-3D	7	WJSC	MTS		
Reproducibility/ precision	Intra-assay variation	WJSC	MTS	12 **	NIH 3T3	MTS		
		WJSC	NRU	12	HepG2	MTS		
	Inter-assay variation	WJSC	MTS	12	NIH 3T3	MTS		
		WJSC	NRU	12	HepG2	MTS		
		WJSC	MTS-3D	7				
	Inter-culture variation ***	WJSC #1	MTS	5				
		WJSC #2	MTS	5				
		WJSC #3	MTS	5				
	Inter-laboratory variation	WJSC	NRU	12	BALB/c-3T3	NRU	In Silico	ICCVAM Lab #1
								ICCVAM Lab #2
								ICCVAM Lab #3
					NHK	NRU	In Silico	ICCVAM Lab #1
								ICCVAM Lab #2
								ICCVAM Lab #3
Correlation to in vivo lethal doses (incl. human)	Agglomerative hierarchical clustering	WJSC	MTS	12/7	NHK	NRU	In Silico	ICCVAM
		WJSC	NRU	12/7	BALB/c-3T3	NRU	In Silico	
		WJSC	MTS-3D	7	LD_50_ rat		In Silico	RC
					LD_Lo_ human		In Silico	MEIC

* Unless otherwise stated, data were derived from in vitro/ex vivo experiments carried out in our lab. ** For 2 out of the 12 chemicals, the assay was repeated in serum-free conditions. *** WJSC #1 and WJSC #2 were derived from heterozygotic twins, and were used at passage 2; WJSC #3 were derived from an independent donor, and were used at passage 6. MSC = mesenchymal stem cells; ADSC = adipose-tissue-derived stem cells; BMSC = bone-marrow-derived stem cells; WJSC = Wharton’s-jelly-derived stem cells.

**Table 3:** Toxicity prediction outcomes as determined by MTS and NRU assays on WJSCs in two- (2D) and three-dimensional (3D) culture environments.

			RC Rat-Only Weight Regression WJSC (mg/kg)			RC Rat-Only Millimole Regression WJSC (mg/kg)		
Chemicals	Hazard Category	RC Rodent (mg/kg)	MTS	NRU	MTS	MTS	NRU	MTS
			2D		3D	2D		3D
*Mercury(II) chloride*	*1*	*1*	*308 ^U^*	*233 ^U^*	*302 ^U^*	*343 ^U^*	*246 ^U^*	*335 ^U^*
Cycloheximide	1	2	38 ^U^	15 ^U^	-	29 ^U^	10 ^U^	-
*Sodium* *arsenite*	*2*	*41*	*139 ^U^*	*126 ^U^*	*139 ^U^*	*88 ^U^*	*78 ^U^*	*89 ^U^*
*Sodium dichromate dihydrate*	*2*	*50*	*72 ^U^*	*32*	*111 ^U^*	*65 ^U^*	*25*	*108 ^U^*
Cadmium(II) chloride	3	88	164 (105) *	356 ^U^	-	135 (77) *	326 ^U^	-
*Sodium* *fluoride*	*3*	*180*	419 ^U^ (274) *	*379 ^U^*	*361 ^U^*	173 (105) *	*154*	*145*
***Propranolol hydrochloride***	***4***	***470***	***488***	***500***	***575***	***619***	***636***	***751***
Atropine sulfate monohydrate	4	639	1593	1373	-	4033 ^U^	2416 ^U^	-
***Potassium chloride***	***5***	***2602***	***2976***	***2981***	***2575***	***2411***	***3386***	***2033***
Trichloroacetic acid	5	4999	1637 ^O^	1344 ^O^	-	1849 ^O^	1466 ^O^	-
*Sodium hypochlorite*	*6*	*10,328*	*1140 ^O^*	*945 ^O^*	*1110 ^O^*	*777 ^O^*	*622 ^O^*	*753 ^O^*
**Glycerol**	**6**	**12,691**	**6401**	**6343**	**-**	**6702**	**6301**	**-**
Correct prediction score (%) **	*N* = 12		5/12 (41.7) [6/12 (50)] *	5/12 (41.7)	-	5/12 (41.7) [5/12 (41.7)] *	5/12 (41.7)	-
	*N* = 7		2/7 (29) 3/7 (42.9)	-	2/7 (29)	3/7 (42.9)	-	3/7 (42.9)
% Prediction improvement	*N* = 12		+9/+17	+9/+17	-	+9/+0	+9/+0	-

^U^ = underprediction (underestimation of toxicity); ^O^ = overprediction (overestimation of toxicity). Chemicals in bold = correct prediction of toxicity class by both regression types (weight/millimole). The 7 chemicals in italics are those selected for confined group analysis based on N = 7 chemicals (one per hazard class; two from class 2) in 2D or 3D culture conditions. * Bracketed data represent predicted LD_50_ values and correct prediction scores resulting from in vitro (2D or 3D) toxicity evaluation in serum-free conditions, using the MTS assay. ** Prediction scores for BALB/c 3T3 and NHK cells (ICCVAM validation study) were 33.3/25% and 33.3/41.7% for the RC-rat only weight and millimole regressions, respectively; % prediction improvement was compared to the available in silico data from the NRU assay for BALB/c 3T3 and NHK cells, respectively.

**Table 4:** Summary of toxicity assay performances.

		LogIC_50_ (mM)–LogLD_50_ (mmol/kg) Linear Regression		Correct Toxicity Prediction Rate (%)	
		r^2^	*p*	RC Rat-Only Weight	RC Rat-Only Millimole
12 chemicals (in vitro/in silico data)	WJSC/WJSC *	0.715/0.775	<0.001	41.7/50	41.7/41.7
	WJSC-NRU	0.695	<0.001	41.7	41.7
	HepG2	0.788	<0.001	41.7	*67*
	NIH 3T3	0.758	<0.001	41.7	33.3
	NHK	0.795	<0.001	25	41.7
	Balb/c-3T3	0.774	<0.001	33.3	33.3
	BMSC	0.628	<0.01	41.7	41.7
7 chemicals (in vitro/in silico data)	WJSC/WJSC *	0.518/0.513	NS/<0.05	28.6/42.9	42.9/42.9
	WJSC-3D	0.590	<0.05	28.6	42.9
	WJSC-NRU	0.482	NS	42.9	42.9
	ADSC	0.538	<0.05	42.9	14.3
	HepG2	0.683	<0.05	42.9	*71.4*
	NIH 3T3	0.607	<0.05	42.9	28.6

*p* = Level of significance, two-tailed value; NS = non-significant (*p* > 0.05) association; * = serum-free conditions; r^2^ = goodness-of-fit coefficient (linear regression).

## Data Availability

Data are available upon reasonable request.

## Supplementary Materials

The following supporting information can be downloaded at: https://www.mdpi.com/article/10.3390/cells11071102/s1, Figure S1: Comparison of experimentally and theoretically determined IC_50_ values between WJSCs and other cell types employed in the study. Toxicity in vitro was estimated via the NRU assay. IC_50_ values for primary cells and cell lines are shown as bars, whereas the theoretical IC_50_ derived from the rat oral LD_50_ values using the RC rat-only weight regression equation is depicted by the solid line.

## References

[1] Hughes J.P., Rees S., Kalindjian S.B., Philpott K.L. (2011). Principles of early drug discovery. Br. J. Pharmacol..

[2] Hartung T. (2017). Evolution of toxicological science: The need for change. Int. J. Risk Assess. Manag..

[3] Meigs L., Smirnova L., Rovida C., Leist M., Hartung T. (2018). Animal testing and its alternatives—The most important omics is economics. ALTEX.

[4] Russell W.M.S., Burch R.L. (1959). The Principles of Humane Experimental Technique.

[5] ICCVAM (2006). Background Review Document: In Vitro Basal Cytotoxicity Test Methods for Estimating Acute Oral Systemic Toxicity. National Institute for Environmental Health Sciences, Research Triangle Park, NC. https://ntp.niehs.nih.gov/iccvam/docs/acutetox_docs/brd_tmer/brdvol1_nov2006.pdf.

[6] ICCVAM (2006). ICCVAM Test Method Evaluation Report. In Vitro Cytotoxicity Test Methods for Estimating Starting Doses for Acute Oral Systemic TOXICITY testing. National Institute of Environmental Health Sciences, Research Triangle Park, NC. https://ntp.niehs.nih.gov/iccvam/docs/acutetox_docs/brd_tmer/at-tmer-complete.pdf.

[7] Cao C., Madren-Whalley J., Kristina C., Valdes J. In Vitro Methods to Measure Toxicity Of Chemicals; 2004, p. 8. https://www.researchgate.net/publication/235041929_In_Vitro_Methods_To_Measure_Toxicity_Of_Chemicals.

[8] Nations U. (2011). Globally Harmonized System of Classification and Labelling of Chemicals (GHS).

[9] Halle W. (2003). The Registry of Cytotoxicity: Toxicity testing in cell cultures to predict acute toxicity (LD50) and to reduce testing in animals. Altern. Lab. Anim..

[10] Gerets H.H.J., Tilmant K., Gerin B., Chanteux H., Depelchin B.O., Dhalluin S., Atienzar F.A. (2012). Characterization of primary human hepatocytes, HepG2 cells, and HepaRG cells at the mRNA level and CYP activity in response to inducers and their predictivity for the detection of human hepatotoxins. Cell Biol. Toxicol..

[11] Hawksworth G.M. (1994). Advantages and disadvantages of using human cells for pharmacological and toxicological studies. Hum. Exp. Toxicol..

[12] Kumar D., Baligar P., Srivastav R., Narad P., Raj S., Tandon C., Tandon S. (2021). Stem Cell Based Preclinical Drug Development and Toxicity Prediction. Curr. Pharm. Des..

[13] Kim T.-W., Che J.-H., Yun J.-W. (2019). Use of stem cells as alternative methods to animal experimentation in predictive toxicology. Regul. Toxicol. Pharmacol..

[14] Scanu M., Mancuso L., Cao G. (2011). Evaluation of the use of human Mesenchymal Stem Cells for acute toxicity tests. Toxicol. Vitr..

[15] Ryu E., Hong S., Kang J., Woo J., Park J., Lee J., Seo J.-S. (2008). Identification of senescence-associated genes in human bone marrow mesenchymal stem cells. Biochem. Biophys. Res. Commun..

[16] Kern S., Eichler H., Stoeve J., Klüter H., Bieback K. (2006). Comparative analysis of mesenchymal stem cells from bone marrow, umbilical cord blood, or adipose tissue. Stem Cells.

[17] Christodoulou I., Goulielmaki M., Devetzi M., Panagiotidis M., Koliakos G., Zoumpourlis V. (2018). Mesenchymal stem cells in preclinical cancer cytotherapy: A systematic review. Stem Cell Res. Ther..

[18] Christodoulou I., Kolisis F.N., Papaevangeliou D., Zoumpourlis V. (2013). Comparative Evaluation of Human Mesenchymal Stem Cells of Fetal (Wharton’s Jelly) and Adult (Adipose Tissue) Origin during Prolonged In Vitro Expansion: Considerations for Cytotherapy. Stem Cells Int..

[19] Spielmann H., Genschow E., Liebsch M., Halle W. (1999). Determination of the Starting Dose for Acute Oral Toxicity (LD50) Testing in the Up and Down Procedure (UDP) From Cytotoxicity Data. Altern. Lab. Anim..

[20] Barltrop J.A., Owen T.C., Cory A.H., Cory J.G. (1991). 5-(3-carboxymethoxyphenyl)-2-(4,5-dimethylthiazolyl)-3-(4-sulfophenyl)tetrazolium, inner salt (MTS) and related analogs of 3-(4,5-dimethylthiazolyl)-2,5-diphenyltetrazolium bromide (MTT) reducing to purple water-soluble formazans As cell-viability indicat. Bioorg. Med. Chem. Lett..

[21] Borenfreund E., Puerner J.A. (1985). Toxicity determined in vitro by morphological alterations and neutral red absorption. Toxicol. Lett..

[22] Ekwall B., Barile F.A., Castano A., Clemedson C., Clothier R.H., Dierickx P., Ekwall B., Ferro M., Fiskesjö G., Garza-Ocañas L. (1998). MEIC Evaluation of Acute Systemic Toxicity: Part VI. The Prediction of Human Toxicity by Rodent LD50 Values and Results From 61 In Vitro Methods. Altern. Lab. Anim..

[23] Ekwall B., Bondesson I., Castell J.V., Gómez-Lechón M.J., Hellberg S., Högberg J., Jover R., Ponsoda X., Romert L., Stenberg K. (1989). Cytotoxicity Evaluation of the First Ten MEIC Chemicals: Acute Lethal Toxicity in Man Predicted by Cytotoxicity in Five Cellular Assays and by Oral LD50 Tests in Rodents. Altern. Lab. Anim..

[24] Clemedson C., Ekwall B. (1999). Overview of the Final MEIC Results: I. The In Vitro–In Vitro Evaluation. Toxicol. Vitr..

[25] Kammerer S., Küpper J.-H. (2018). Human hepatocyte systems for in vitro toxicology analysis. J. Cell. Biotechnol..

[26] Abud A.P.R., Paschoal A.C.C., Kuligovski C., Caruso R.R.B., Dallagiovanna B., de Aguiar A.M. (2021). Using inhibition of the adipogenesis of adipose-derived stem cells in vitro for toxicity prediction. MethodsX.

[27] Abud A.P.R., Zych J., Reus T.L., Kuligovski C., de Moraes E., Dallagiovanna B., de Aguiar A.M. (2015). The use of human adipose-derived stem cells based cytotoxicity assay for acute toxicity test. Regul. Toxicol. Pharmacol..

[28] Larsson P., Engqvist H., Biermann J., Werner Rönnerman E., Forssell-Aronsson E., Kovács A., Karlsson P., Helou K., Parris T.Z. (2020). Optimization of cell viability assays to improve replicability and reproducibility of cancer drug sensitivity screens. Sci. Rep..

[29] Zhang R., Zhang H., Chen B., Luan T. (2020). Fetal bovine serum attenuating perfluorooctanoic acid-inducing toxicity to multiple human cell lines via albumin binding. J. Hazard. Mater..

[30] Zhang Y., Xu Y.-Y., Sun W.-J., Zhang M.-H., Zheng Y.-F., Shen H.-M., Yang J., Zhu X.-Q. (2016). FBS or BSA Inhibits EGCG Induced Cell Death through Covalent Binding and the Reduction of Intracellular ROS Production. Biomed. Res. Int..

[31] Thomas M.G., Marwood R.M., Parsons A.E., Parsons R.B. (2015). The effect of foetal bovine serum supplementation upon the lactate dehydrogenase cytotoxicity assay: Important considerations for in vitro toxicity analysis. Toxicol. Vitr..

[32] Aslantürk Ö. (2018). In Vitro Cytotoxicity and Cell Viability Assays: Principles, Advantages, and Disadvantages.

[33] Goodwin C.J., Holt S.J., Downes S., Marshall N.J. (1995). Microculture tetrazolium assays: A comparison between two new tetrazolium salts, XTT and MTS. J. Immunol. Methods.

[34] Riss T.L., Moravec R.A. (1992). Comparison of MTT, XTT, and a novel tetrazolium compound MTS for in vitro proliferation and chemosensitivity assays. Mol. Biol. Cell.

[35] Zurita J.L., Jos Á., del Peso A., Salguero M., López-Artíguez M., Repetto G. (2005). Ecotoxicological evaluation of the antimalarial drug chloroquine. Aquat. Toxicol..

[36] Zuang V. (2001). The neutral red release assay: A review. Altern. Lab. Anim..

[37] Clothier R., Gómez-Lechón M.J., Kinsner-Ovaskainen A., Kopp-Schneider A., O’Connor J.-E., Prieto P., Stanzel S. (2012). Comparative analysis of eight cytotoxicity assays evaluated within the ACuteTox Project. Toxicol. Vitr..

[38] Nicolas J., Magli S., Rabbachin L., Sampaolesi S., Nicotra F., Russo L. (2020). 3D Extracellular Matrix Mimics: Fundamental Concepts and Role of Materials Chemistry to Influence Stem Cell Fate. Biomacromolecules.

[39] Jensen C., Teng Y. (2020). Is It Time to Start Transitioning From 2D to 3D Cell Culture?. Front. Mol. Biosci..

[40] Langhans S.A. (2018). Three-Dimensional in Vitro Cell Culture Models in Drug Discovery and Drug Repositioning. Front. Pharmacol..

