# Supplementary figures and images for: Suitability of Human Mesenchymal Stem Cells Derived from Fetal Umbilical Cord (Wharton’s Jelly) as an Alternative In Vitro Model for Acute Drug Toxicity Screening
Source: Cells. 2022 Mar 24;11(7):1102. doi: 10.3390/cells11071102 (PMC8997545; doi:10.3390/cells11071102)

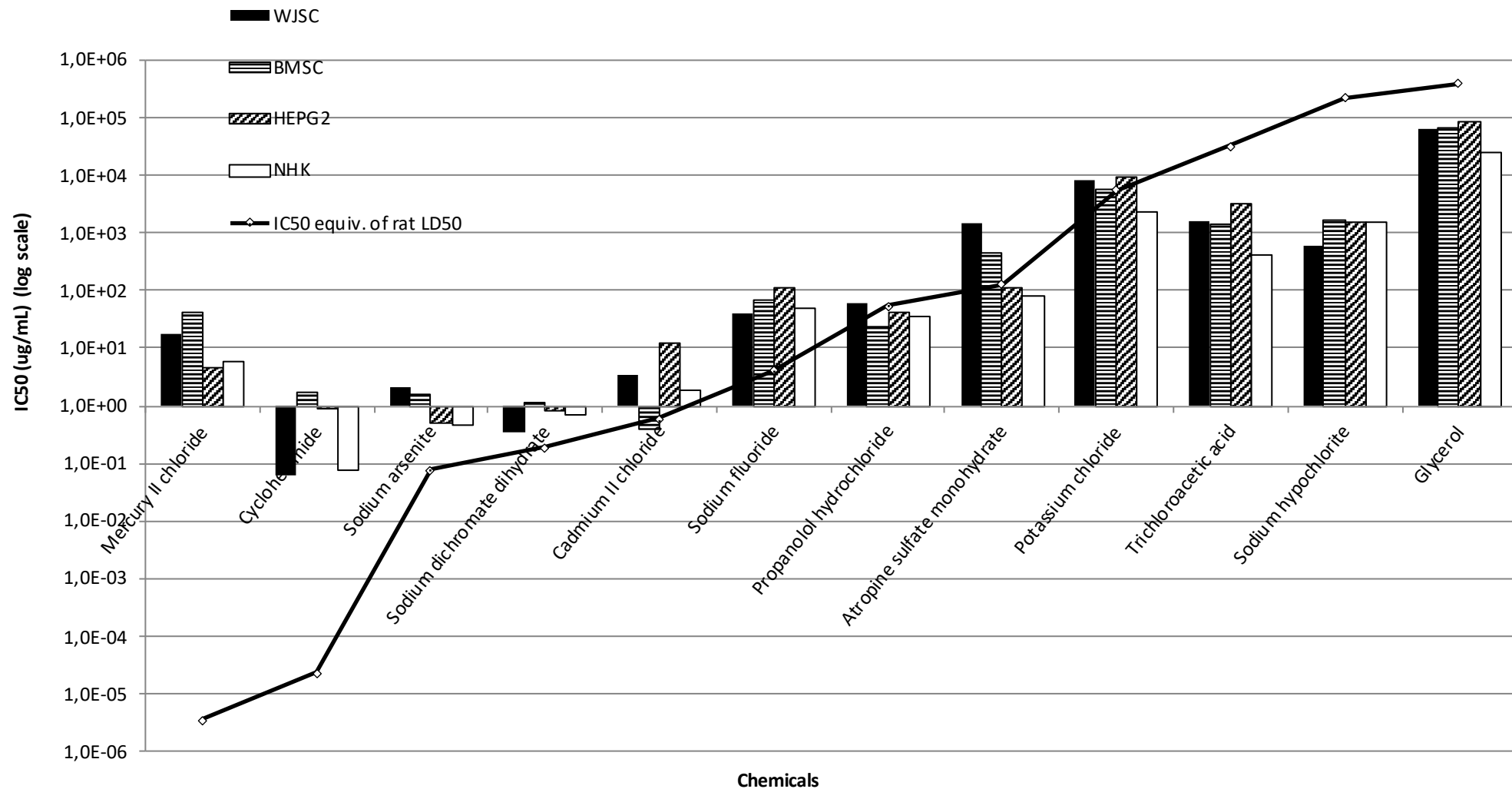

Supplement: Supplementary file 1 [file cells-11-01102-s001.zip › cells-1582851-supplementary.pdf]
